# Supplementary material for: Development and validation of AI/ML derived splice-switching oligonucleotides
Source: Mol Syst Biol. 2024 Apr 25;20(6):676–701. doi: 10.1038/s44320-024-00034-9 (PMC11148135; doi:10.1038/s44320-024-00034-9)
Supplement: Supplementary file 1 — Appendix [file 44320_2024_34_MOESM1_ESM.pdf]

Development and validation of AI/ML derived splice-switching oligonucleotides

Alyssa D Fronk[1,†], Miguel A Manzanares[1,†], Paulina Zheng[1], Adam Geier[1], Kendall Anderson[1], Shaleigh Stanton[1], Hasan Zumrut[1], Sakshi Gera[1], Robin Munch[1], Vanessa Frederick[1], Priyanka Dhingra[1], Gayatri Arun[1], and Martin Akerman[1,*]
[†]Equal contribution
*To whom correspondence should be addressed. Email: makerman@envisagenics.com

Affiliations
[1] Envisagenics, Inc., Long Island City, NY 11101

Table of Contents

Appendix Supplementary Methods.. 2
The SpliceCore® platform 2
TXdb Assembly 2
Appendix Table S1. Predictive performance (AUC values) of six different AI/ML types for upstream intronic, exonic and downstream intronic models.. 4
Appendix Figure S1. Validation of most predictive SFs. 5
Appendix Figure S2. Splicing factors alterations in triple negative breast cancer. 6
Appendix Figure S3. Benchmarks of SpliceCore's RNA-seq analysis. 7
Appendix Figure S4. TXdb exon centric reference transcriptome assembly 9
Appendix Table S2. List of SSO sequences..10
Appendix Figure S5. SSOs targeting downstream intron do not affect NEDD4Le13 splicing.....11
Appendix Figure S6. NEDD4Le13 skipping modulates the degradation of TGFβ pathway-related proteins by the proteasome...12
Appendix Supplemental References 13

Appendix Supplementary Methods

The SpliceCore® platform

SpliceCore takes RNA-seq FASTQ/A files as an input to produce alternative splicing (AS) profiles between "case" and "control" datasets. AS analysis is often divided into three steps: alignment, quantification, and comparison (Alamancos *et al*, 2014) (Appendix Figure S3A-B). The SpliceCore platform uses the SpliceTrap algorithm (Wu *et al*, 2011) to align RNA-seq data to TXdb and quantify the "percent spliced in" (PSI) of every AS event. Next, SpliceDuo performs case/control comparisons and reports splicing changes as ΔPSI values between -100% (i.e., full exon skipping) and 100% (full exon inclusion) (Anczuków *et al*, 2015).

Sequence alignment is the most time-consuming step of RNA-seq analysis, in part due to the use of a large reference transcriptome like TXdb, with 1,743,426 AS annotations (see TXdb assembly section). However, most RNA-seq analysis projects only require a single alignment iteration. One-time sequence alignment is common practice in bioinformatics, as it is supported by evidence that changes in alignment parameters have little impact on both technical and biological performance (Ballouz *et al*, 2018). In contrast, the comparison step is often repeated multiple times to allow thorough interrogation of the data (Appendix Figure S3A-B). This is especially important when analyzing RNA-seq data from heterogeneous patient cohorts, including subjects with various disease subtypes, at different disease stages, responding differently to drug treatments, and with diverse clinical backgrounds. The motivation to perform multiple comparisons has only increased as a result of progress in the areas of personalized therapies and discovery of biomarkers (Shyr & Liu, 2013). The SpliceTrap algorithm is optimized for a one-time execution step that includes sequence alignment and PSI quantification. Some of the most popular tools for AS analysis, like rMATS (Shen *et al*, 2014), MAJIQ (Green *et al*, 2018), and MISO (Katz *et al*, 2010), offer a combined solution for quantification and comparison across pre-aligned BAM files generated with other tools such as STAR aligner (Dobin *et al*, 2013) (Appendix Figure S3B). While these are all highly accurate tools for AS analysis, they carry the burden of unnecessary repeated quantifications, when only the comparisons should be repeated. To expedite data analysis, SpliceCore offers an alternative software design, by pooling one-time alignment and quantification with SpliceTrap, and allowing for fast and scalable statistical modeling of AS comparison using SpliceDuo. Benchmarking to open-source competitors demonstrated that SpliceCore performs in a significantly larger search space (Appendix Figure S3C) with outstanding speed (Appendix Figure S3D-G), scalability (Appendix Figure S3H-J), and accuracy (Appendix Figure S3K-O), thereby accelerating value extraction from RNA-seq data.

TXdb Assembly

TXdb is an exon-centric reference transcriptome used by all of SpliceCore's algorithms (Wu *et al*, 2011). The premise of "exon-centric" is to treat the transcriptome as a collection of independent AS events rather than full-length transcripts. In TXdb, CA and IR events are represented as exon trios where the middle exon is subjected to AS analysis and the flanking exons provide the transcriptomic context with the corresponding splicing junctions necessary for the analysis. Every exon trio is presented in two splicing states: inclusion, where the three exons are connected through a pair of splicing junctions, and skipping, where the flanking exons are connected by a single splicing junction and the middle exon is skipped. Likewise, alternative 3' splice sites (A3SS) and alternative 5' splice sites (A5SS) are represented as exon duos where the extended exon segment is subjected to AS analysis and the remaining sequences provide context (Appendix Figure S4A). For simplicity, we use the term "inclusion" to also define intron retention and distal A3SS and A5SS splicing; and we use "skipping" to define intron splicing and proximal A3SS and A5SS splicing. In addition, CA events in TXdb include annotations for both alternative and constitutive exons, i.e., for exon trios only supported by their inclusion state. In contrast, evidence for both inclusion and skipping states were required for IR, A3SS and A5SS as a way to limit the

sequence search space due to lengthy introns and the exponentially large number of dinucleotides matching the splice site consensus that could result in A3SS or A5SS. To generate TXdb, we assembled mRNA contigs from 1,252 breast cancer RNA-seq datafiles from TCGA. STAR aligner (Dobin *et al*, 2013) was used for read mapping and Stringtie (Pertea *et al*, 2015) for contig building. A total of 5,617,407 AS events were supported by at least one out of 1,252 RNA-seq datafiles, although many of these AS events were redundant. Almost every AS was clustered with several others showing identical inclusion and skipping junctions, identical middle (or extended) exon, but flanking exons of different sizes. We pruned redundant clusters of AS events by selecting a single representative exon trio/duo by applying several prioritization steps. First, we looked for known AS events supported by Ensemble or RefSeq. Second, we prioritized exon trios/duos supported by Ensemble or RefSeq. Third, we took AS events with the highest reliability score. Finally, if a tie persisted, we selected the shortest AS event to further reduce sequence search space (Appendix Figure S4B). We identified a total of 1,743,426 non-redundant AS events, including 1,190,514 CA, 199,238 IR, 202,851 A3SS and 150,823 A5SS (Appendix Figure S4C). Also 534,231 (31%) exon trios/duos were supported by ENSEMBL or RefSeq (GRCh38.p12), 77,381 (4%) further presented both inclusion and skipping evidence in ENSEMBL or RefSeq (i.e. known AS events). The remaining 1,131,814 (65%) showed no evidence of in public mRNA databases and were therefore annotated as novel trios/duos (Appendix Figure S4D) (Cheung *et al*, 2019; Lambert *et al*, 2014; Ray *et al*, 2013; Paz *et al*, 2014).

Appendix Table S1.

	up intron	exon	down intron
XGBoost	0.88	0.60	0.95
Random Forest	0.80	0.65	0.92
GradientBoost	0.78	0.64	0.92
AdaBoost	0.73	0.65	0.93
Logistic Regression	0.73	0.61	0.92
SVM	0.67	0.49	0.92

Appendix Table S1. Predictive performance (AUC values) of six different AI/ML types for upstream intronic, exonic and downstream intronic models

Appendix Figure S1.

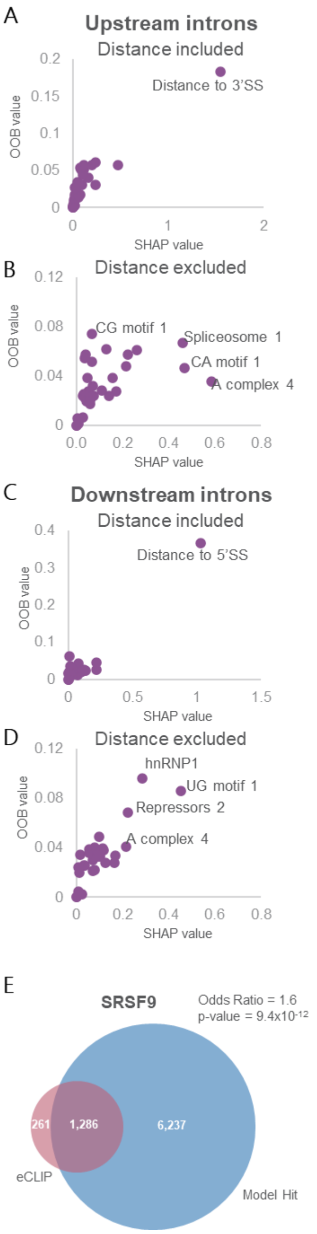

F

SF	Model Hit and eCLIP	eCLIP	Model Hit	No hits	Odds ratio	p-value
SF3B4	7166	5423	18001	18547	1.361489	5.43E-50
QKI	585	3920	1866	13277	1.061838	0.237548
HNRNPL	3055	3372	9948	11296	1.028756	0.325083
SFPQ	13	1308	84	5490	0.649574	0.192494
U2AF2	5450	6002	24327	34592	1.291182	1.53E-35
SRSF9	1286	261	6273	2052	1.611768	9.43E-12
SRSF7	809	1791	3424	8883	1.171868	0.000803
PCBP2	869	1339	3018	4496	0.966821	0.504734
SRSF1	77	3449	251	14829	1.318972	0.039083
IGF2BP3	567	1573	2291	6524	1.026463	0.640892
PABPC4	275	98	2120	885	1.171424	0.226705
PCBP1	0	66	4	434	0	1

G

SF	Weighted AS effect probability	SC in top X% of SHAP values	SF percentile score cutoff	Odds ratio	p-value
HNRNPL	0.9	0.5	50	1.193732	0.215645
SRSF1	0.9	0.25	75	8.438503	0.059118
SF3B4	0.9	0.5	50	4.576	1.20E-61
U2AF2	0.8	0.5	50	1.42856	0.033671
SRSF7	0.9	0.5	50	2.098265	4.45E-05
QKI	0.9	0.25	50	1.515991	0.124184
HNRNPC	0.5	0.5	75	1.036879	0.896245
SFPQ	0.9	0.5	50	1.387785	0.288152
PTBP1	0.95	0.5	50	4.754237	0.002987
HNRNPK	0.7	0.5	50	5.318436	0.000885
SRSF9	0.6	0.5	50	2.386817	7.38E-42
PCBP2	0.9	0.25	75	2.618557	0.011713
PCBP1	0.5	0.5	75	1.572425	6.71E-11
IGF2BP3	0.8	0.25	75	1.37931	0.169568
PABPC4	0.9	0.25	75	3.354167	0.044891

Appendix Figure S1. Validation of most predictive SFs. A-D. Scatterplots comparing the weights of the contribution of each feature to the upstream intron model including distance as a feature (A), the upstream intron model excluding distance as a feature (B), the downstream intron model including distance as a feature (C), or the downstream model excluding distance as a feature (D). E. Venn diagram depicting the overlap of positions where SRSF9 is predicted to have an eCLIP peak in the ENCODE datasets and where it is predicted to regulate splicing via XGboost. Odds ratio and p-values were calculated using a Fisher's Exact test. F. Table representing the contingency table for each SF with both XGboost and eCLIP data where the ontology was in the top 25% of features and the XGboost probability is at least 0.5. G. Table representing the cutoff points for XGboost probability (splicing effect probability), SHAP top percent, and SF percentile score cutoff that optimizes for the best odds ratio and p-value as calculated by a Fisher's exact test.

Appendix Figure S2.

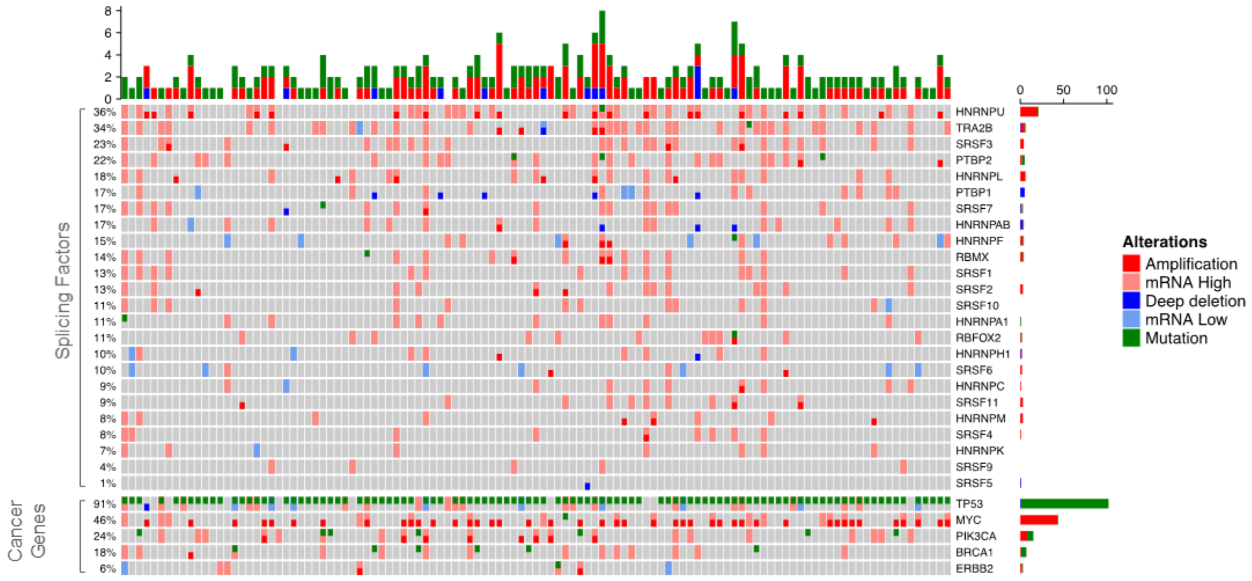

Appendix Figure S2. Splicing factors alterations in triple negative breast cancer. Oncoprint of alterations in activators, repressors, and breast cancer genes in TNBC tumor samples from TCGA. Individual SFs are rows and tumor samples are columns. Number of alterations in each tumor sample and gene is represented in bar plots at the top and right respectively.

Appendix Figure S3.

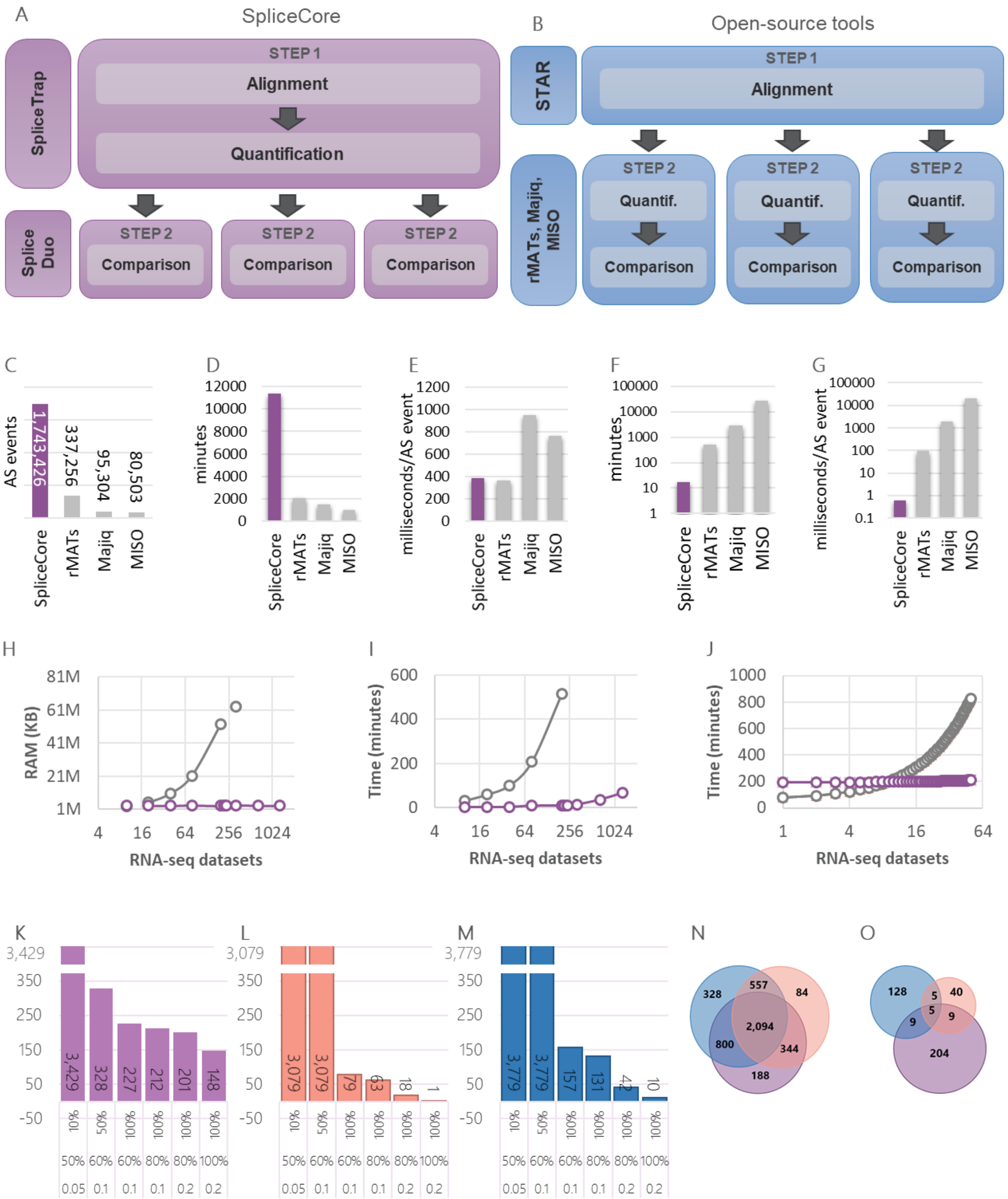

Appendix Figure S3. Benchmarks of SpliceCore's RNA-seq analysis. A, B. RNA-seq analysis workflow of (A) SpliceCore vs. (B) competitive tools. While Step 1 runs only once in every tool, step 2 is often repeated many times, depending on the number of RNA-seq files. C. Number of AS events in the reference transcriptome analyzed by each tool. SpliceCore used TXdb, rMATS

used a GFF index file downloaded from the rMATS website, Majiq used STAR aligner and the Majiq builder tool to build lsv files from the raw RNA-seq files and MISO used alternative events annotations in GFF format from the MISO website. D-G. Run time for (D) step 1 and (F) a single iteration of step 2 are shown along with run times normalized by the total number of interrogated AS events in the reference transcriptome for both (E) step 1 and (G) a single step 2. A total of 82 RNA-seq files were analyzed using 16 CPU and 64 GB RAM. D. SpliceCore takes longer to run step 1 compared to other tools. (F) However, it reaches similar speed to the rMATS/STAR pipeline when run time is normalized by the reference transcriptome size. In contrast, (F,G) SpliceCore is clearly faster than other tools at step 2. H-J. We further compared SpliceCore and rMATS in terms of their ability to analyze increasing amounts of RNA-seq files in step 2 using 16 CPU and 64 GB RAM. (H) SpliceCore was memory-efficient in handling up to 1312 RNA-seq files (i.e., comparing 656 case vs 656 control RNA-seq files). In contrast, rMATS encountered overflow when handling 200 files (100 case vs 100 control), (I) which was also reflected in the overall runtime, demonstrating that only SpliceCore can efficiently integrate several RNA-seq files in step 2. (J) We then performed a single step 1 combined with increasing numbers of step 2 iterations, using both SpliceCore and rMATS on 82 RNA-seq files, 16 CPU and 64 GB RAM. We observed SpliceCore outperforms rMATS when more than 10 step 2 iterations are performed. K-O. We finally quantified the ability of (K) SpliceCore, (L) rMATS and (M) MISO to identify protein-supported AS events annotated in the Uniprot database using varying filters for reproducibility, consistency, and PSI values. The reproducibility filter is defined as the minimum percent of RNA-seq files in which significant AS events should be detected. Consistency is the minimal percent of RNA-seq files showing an AS event in the same direction (i.e., inclusion or skipping). The PSI filter selects AS events above a given "percent-splice-in" value. It can be observed that SpliceCore identified a larger number of AS events with outstanding reproducibility and consistency. (N,O) Venn diagram showing the overlap of AS events detected by SpliceCore (purple), rMATs (blue), and MISO (orange). (N) Filter set at Reproducibility ≥ 10%, Consistency≥50% and PSI ≥ 0.05. (O) Filter set at Reproducibility ≥ 100%, Consistency≥60% and PSI ≥ 0.1

Appendix Figure S4.

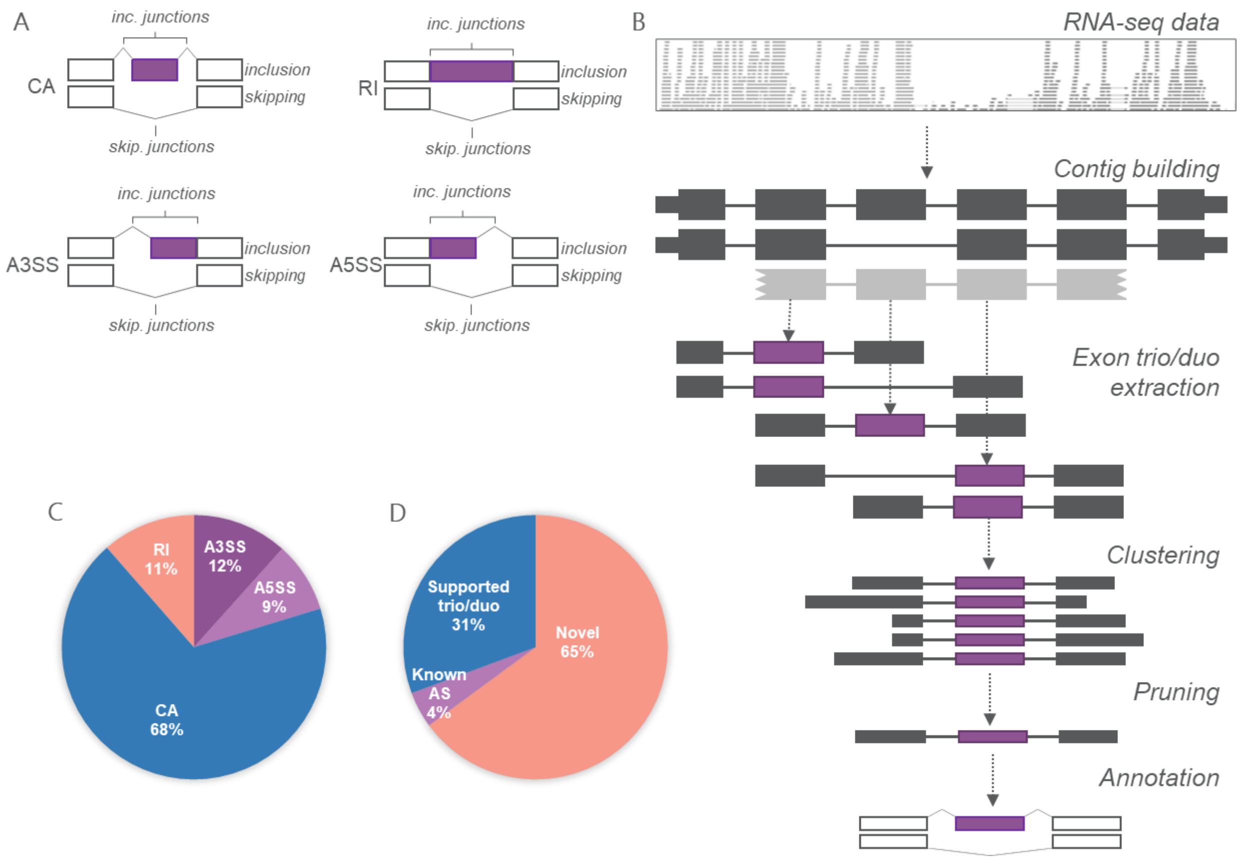

Appendix Figure S4. TXdb exon centric reference transcriptome assembly. A. Four different types of AS events. Cassette exons (CA) and retained introns (IR) are represented as exon trios. Alternative 3' and 5' splice sites (A3SS and A5SS) are represented as exon duos. B. Assembly of TXdb. RNA-seq data from TCGA-BRCA was aligned to the genome using STAR and RNA contigs were constructed using StringTie. Both full (black) and partial (gray) contigs were kept in the pipeline. Next, every observed combination of three or two exons was derived from genomically colocalized contig blocks. Redundant exon trios/duos were clustered and pruned to a single representative. Finally, each AS event was annotated in their inclusion and skipping states to be used as a transcriptomic reference. C. Distribution of different AS types in TXdb. Cassette exons (blue), retained intron (orange), alternative 3' (dark purple) and 5' (light purple) splice sites. D. Exon trios/duos assemblies supported in ENSEMBL or RefSeq as constitutive (blue), subset of known AS events with both inclusion/skipping support (purple) and novel exon trio/duo assemblies (orange).

Appendix Table S2

ID	SpliceLearn output seq	Back bone	Internal	Reverse complement DNA	SSO-seq	
SSO-0201	GCTGGCTTTGTCTGGATAGG	PS	2'MOE	CCTATCCAGACAAAGCCAGC	CCUAUCCAGACAAAGCCAGC	
SSO-0202	GTGGGTTTCAGGGATTCTGA	PS	2'MOE	TCAGAATCCCTGAAACCCAC	UCAGAAUCCCUGAAACCCAC	
SSO-0203	TCTCACGTCACCTGCCTTAC	PS	2'MOE	GTAAGGCAGGTGACGTGAGA	GUAAGGCAGGUGACGUGAGA	
SSO-0204	AGCGCTGCCACAGCAGTGGG	PS	2'MOE	CCCACTGCTGTGGCAGCGCT	CCCACUGCUGUGGCAGCGCU	
SSO-0205	CCCTGATTCAGACAGCAGGG	PS	2'MOE	CCCTGCTGTCTGAATCAGGG	CCCUGCUGUCUGAAUCAGGG	
SSO-0206	GATCTCACGTCACCTGCCTTA	PS	2'MOE	TAAGGCAGGTGACGTGAGATC	UAAGGCAGGUGACGUGAGAUC	
SSO-0207	ATCTCACGTCACCTGCCTTACA	PS	2'MOE	TGTAAGGCAGGTGACGTGAGAT	UGUAAGGCAGGUGACGUGAGAU	
SSO-0208	UACACGAAAUAUUAAAACUG	PS	2'MOE	CAGTTTTAATATTTCGTGTA	CAGUUUUAAUAUUUCGUGUA	NEGATIVE SSOs
SSO-0209	CGUAUAUUCACCACUACACG	PS	2'MOE	CGTGTAGTGGTGAATATACG	CGUGUAGUGGUGAAUAUACG	
SSO-0210	AUUUUUAAAAACAGCGUAUA	PS	2'MOE	TATACGCTGTTTTTAAAAAT	UAUACGCUGUUUUUAAAAAU	
SSO-0211	AAGGAGCUAAUCUUAUUUUU	PS	2'MOE	AAAAATAAGATTAGCTCCTT	AAAAAUAAGAUUAGCUCCUU	
SSO-0212	AGCUCUGUAGCACAAAGGAG	PS	2'MOE	CTCCTTTGTGCTACAGAGCT	CUCCUUUGUGCUACAGAGCU	
SSO-0213	UAAGAAACGUGUGCAGCUCU	PS	2'MOE	AGAGCTGCACACGTTTCTTA	AGAGCUGCACACGUUUCUUA	
SSO-0214	UGAGAAGCUUAAAAUAAGAA	PS	2'MOE	TTCTTATTTTAAGCTTCTCA	UUCUUAUUUUAAGCUUCUCA	

Appendix Table S2. List of SSO sequences

Appendix Figure S5

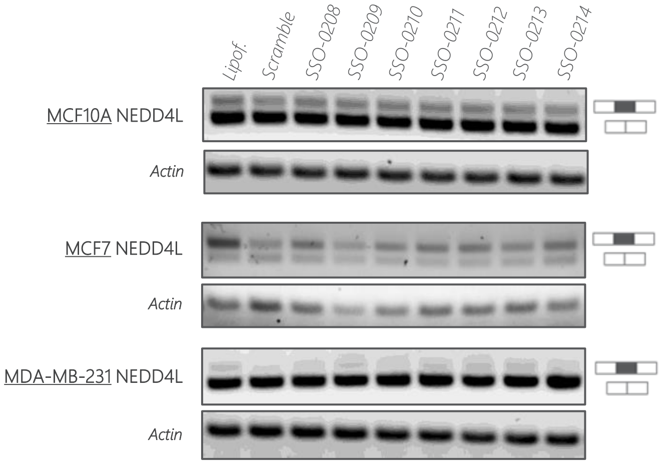

Appendix Figure S5. SSOs targeting downstream intron do not affect NEDD4Le13 splicing. Representative 2% agarose gel for *NEDD4L* PCR showing NEDD4Le3 splicing not been affected by the SSOs treatment (400nM) in three breast tissue cell lines treated for 48h. Actin expression used as cDNA internal control.

Appendix Figure S6.

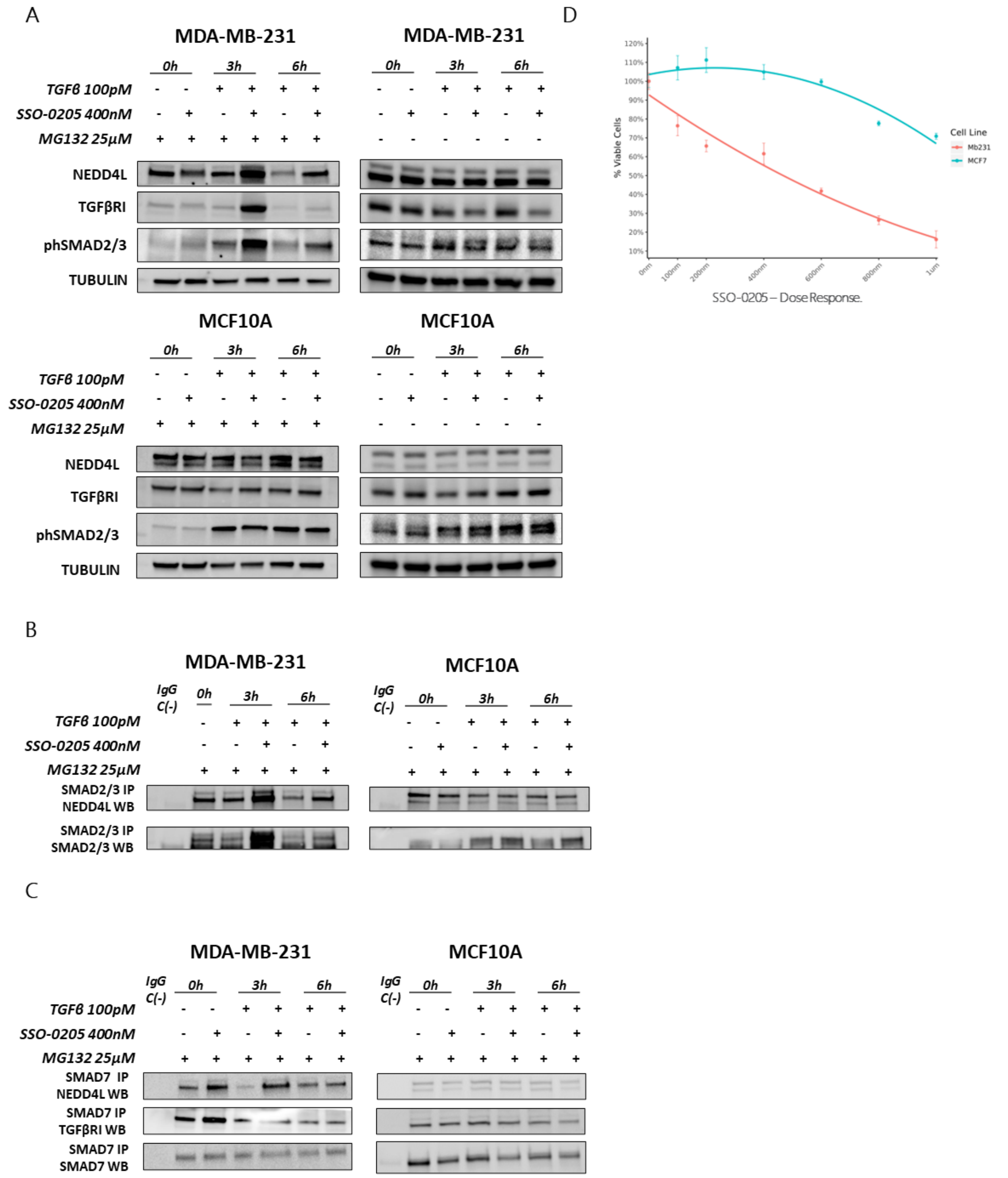

Appendix Figure S6. NEDD4Le13 skipping modulates the degradation of TGFβ pathway-related proteins by the proteasome. A. Western blot measuring TGFβ-pathway related proteins in total lysates in response to TGFβ stimulation (0, 3 or 6 hours) after SSO-0205 treatment (400 nM) (24h) in MDA-MB-231(top panel) and MCF10A cells (lower panel). On the right panels prior to TGFβ treatment proteasome activity was inhibited by treating the cells with MG132 (25 µM). MG132 untreated controls are shown on the right panel. The same protein samples were immunoprecipitated with SMAD2/3 (B) or SMAD7 (C) and Western blots performed for NEDD4L, SMAD2/3, TGFβRI or SMAD7. IgG Negative Control (IgG C(-)) with no cell lysate but subjected to the IP and WB protocol were run to determine nonspecific IgG heavy and light chain contamination. In D Cell TiterGlo® 96 well assay was used to determine MDA-MB-231 and MCF7 cell viability after 48h treatment with SSO-0205 in a dose dependent manner (100nM, 200nM, 400nM, 600nM, 800nM, 1uM).

Appendix Supplemental References

Alamancos GP, Agirre E & Eyras E (2014) Methods to study splicing from high-throughput RNA sequencing data. *Methods Mol Biol* 1126: 357–397

Anczuków O, Akerman M, Cléry A, Wu J, Shen C, Shirole NH, Raimer A, Sun S, Jensen MA, Hua Y, *et al* (2015) SRSF1-Regulated Alternative Splicing in Breast Cancer. *Mol Cell* 60: 105–117

Ballouz S, Dobin A, Gingeras TR & Gillis J (2018) The fractured landscape of RNA-seq alignment: the default in our STARs. *Nucleic Acids Res* 46: 5125–5138

Cheung R, Insigne KD, Yao D, Burghard CP, Wang J, Hsiao YHE, Jones EM, Goodman DB, Xiao X & Kosuri S (2019) A Multiplexed Assay for Exon Recognition Reveals that an Unappreciated Fraction of Rare Genetic Variants Cause Large-Effect Splicing Disruptions. *Mol Cell* 73: 183-194.e8

Dobin A, Davis CA, Schlesinger F, Drenkow J, Zaleski C, Jha S, Batut P, Chaisson M & Gingeras TR (2013) STAR: ultrafast universal RNA-seq aligner. *Bioinformatics* 29: 15–21

Green CJ, Gazzara MR & Barash Y (2018) MAJIQ-SPEL: web-tool to interrogate classical and complex splicing variations from RNA-Seq data. *Bioinformatics* 34: 300–302

Katz Y, Wang ET, Airoldi EM & Burge CB (2010) Analysis and design of RNA sequencing experiments for identifying isoform regulation. *Nat Methods* 7: 1009–1015

Lambert N, Robertson A, Jangi M, McGeary S, Sharp PA & Burge CB (2014) RNA Bind-n-Seq: Quantitative Assessment of the Sequence and Structural Binding Specificity of RNA Binding Proteins. *Mol Cell* 54: 887–900

Paz I, Kosti I, Ares M, Cline M & Mandel-Gutfreund Y (2014) RBPmap: A web server for mapping binding sites of RNA-binding proteins. *Nucleic Acids Res* 42: 1–7

Pertea M, Pertea GM, Antonescu CM, Chang TC, Mendell JT & Salzberg SL (2015) StringTie enables improved reconstruction of a transcriptome from RNA-seq reads. *Nat Biotechnol* 33: 290–295

Ray D, Kazan H, Cook KB, Weirauch MT, Najafabadi HS, Li X, Gueroussov S, Albu M, Zheng H, Yang A, *et al* (2013) A compendium of RNA-binding motifs for decoding gene regulation. *Nature* 499: 172

Shen S, Park JW, Lu ZX, Lin L, Henry MD, Wu YN, Zhou Q & Xing Y (2014) rMATS: Robust and flexible detection of differential alternative splicing from replicate RNA-Seq data. *Proc Natl Acad Sci U S A* 111: E5593–E5601

Shyr D & Liu Q (2013) Next generation sequencing in cancer research and clinical application. *Biol Proced Online* 15

Wu J, Akerman M, Sun S, McCombie WR, Krainer AR & Zhang MQ (2011) SpliceTrap: a method to quantify alternative splicing under single cellular conditions. *Bioinformatics* 27: 3010–3016
