# Supplementary figures and images for: Development and validation of AI/ML derived splice-switching oligonucleotides
Source: Mol Syst Biol. 2024 Apr 25;20(6):676–701. doi: 10.1038/s44320-024-00034-9 (PMC11148135; doi:10.1038/s44320-024-00034-9)

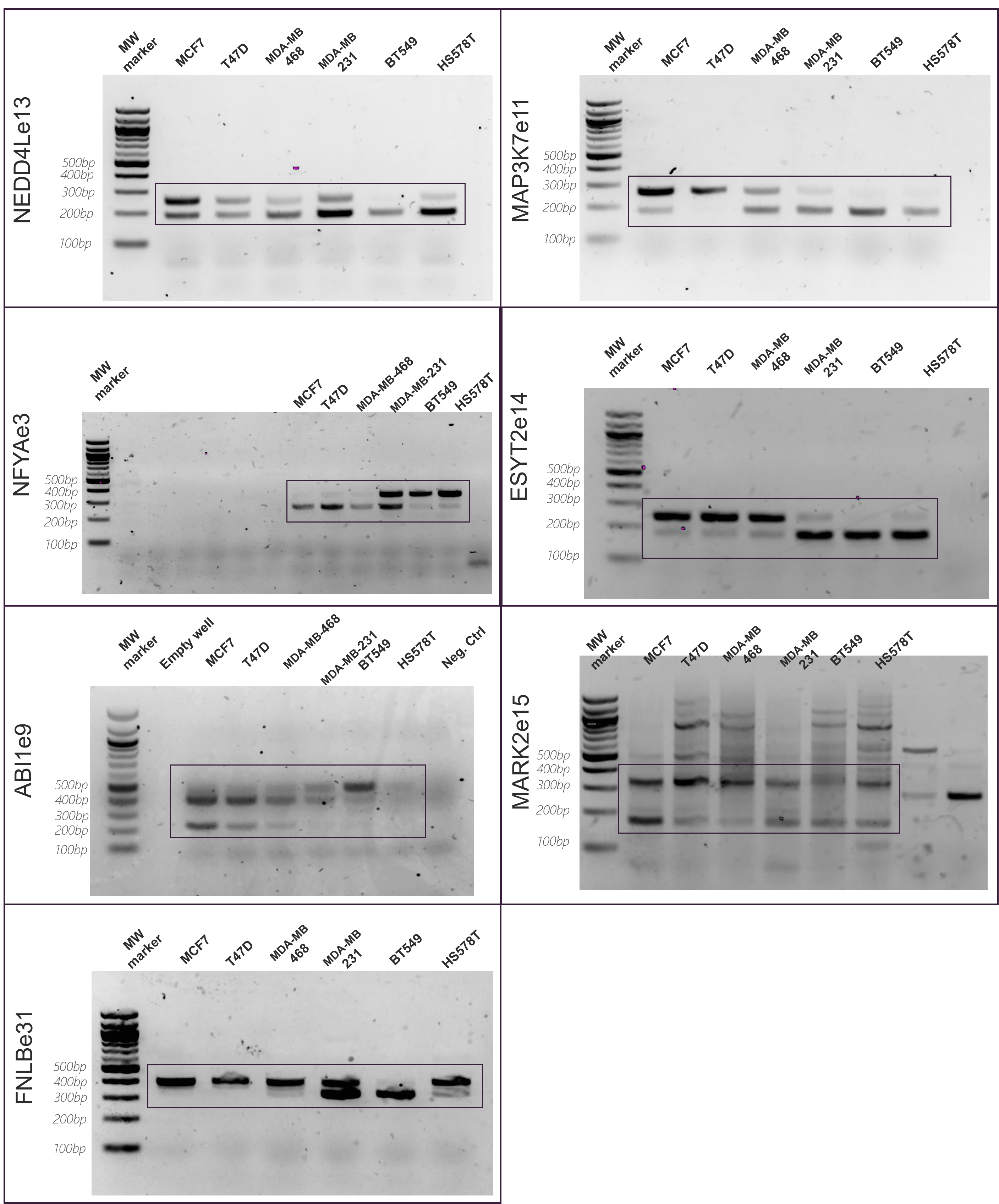

Supplement: Supplementary file 6 — Source data Fig. 3 [file 44320_2024_34_MOESM6_ESM.zip › MSB-2023-12002R_SourceDataForFigure3C.tif]

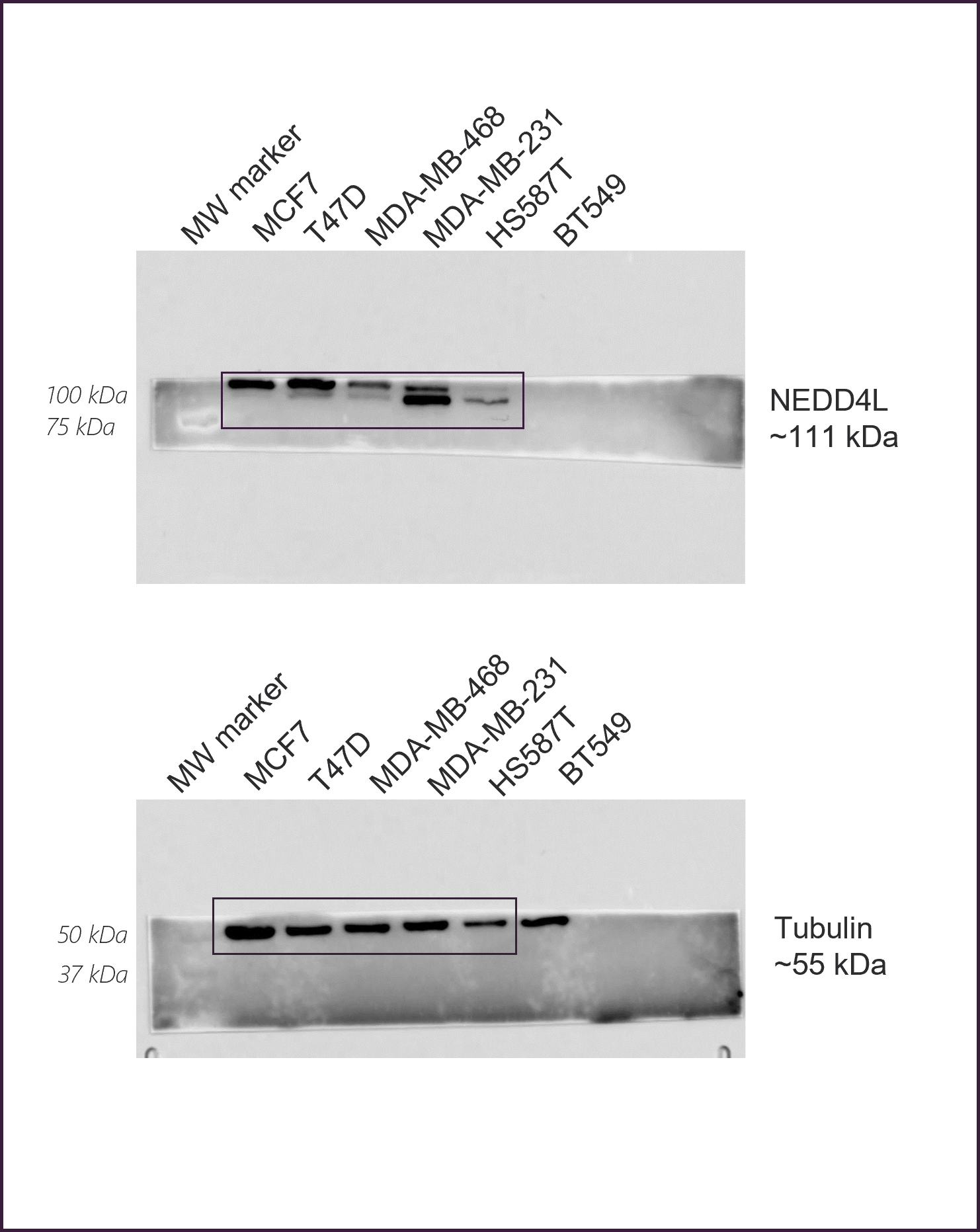

Supplement: Supplementary file 7 — Source data Fig. 4 [file 44320_2024_34_MOESM7_ESM.zip › MSB-2023-12002R_SourceDataForFigure4A.tif]

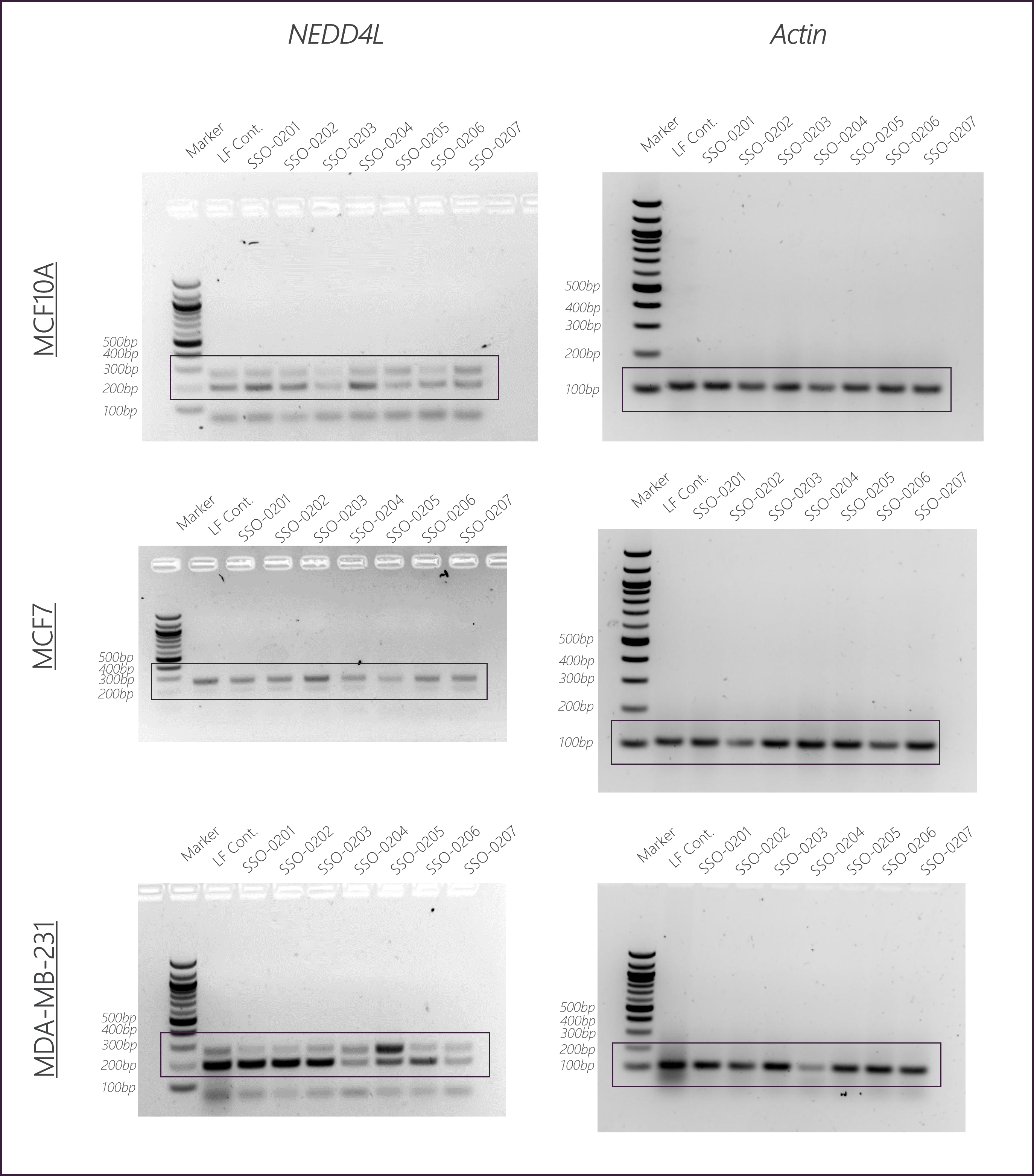

Supplement: Supplementary file 8 — Source data Fig. 6 [file 44320_2024_34_MOESM8_ESM.zip › MSB-2023-12002R_SourceData_Figure6/MSB-2023-12002R_SourceData Figure6A.tif]

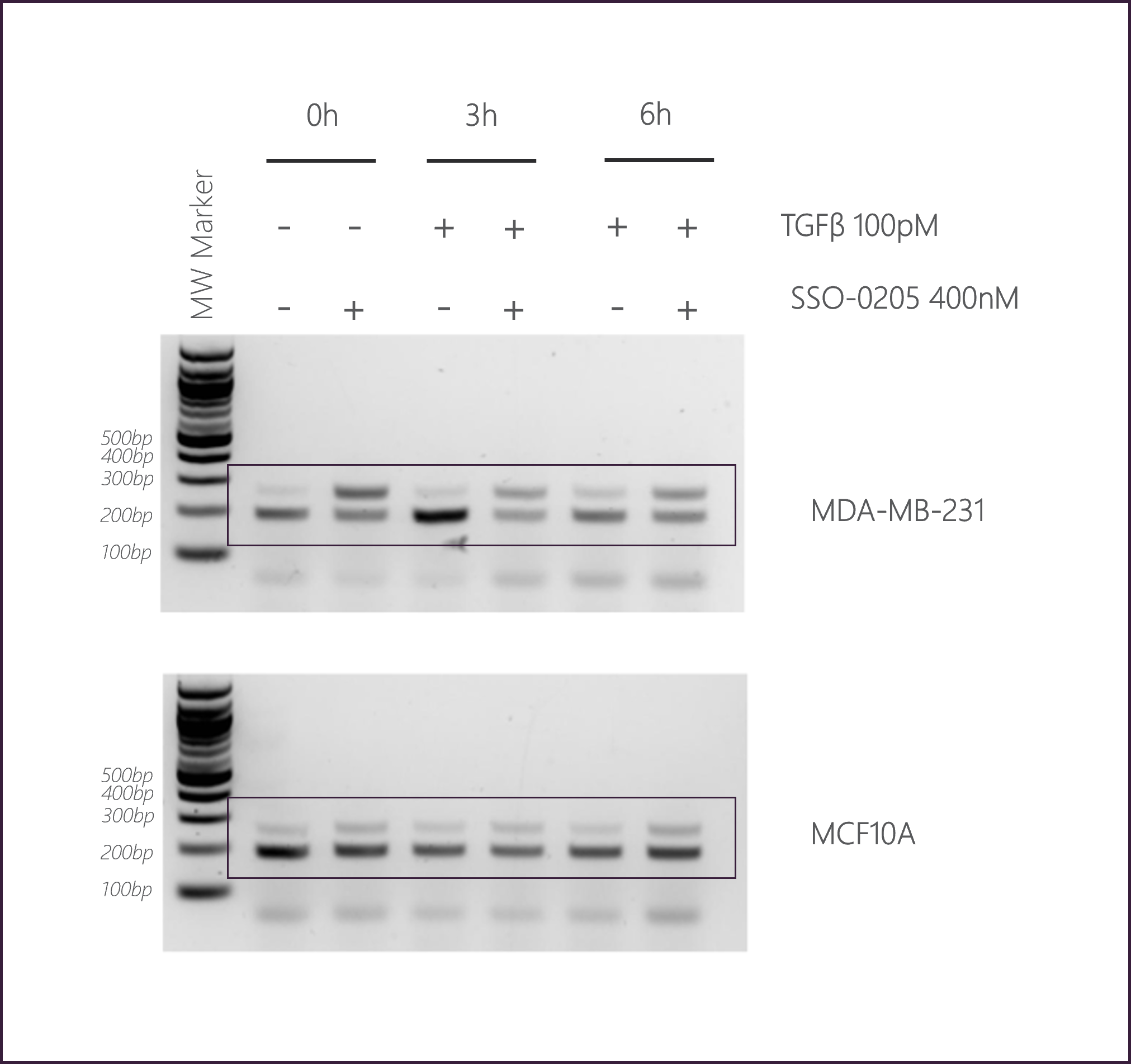

Supplement: Supplementary file 9 — Source data Fig. 7 [file 44320_2024_34_MOESM9_ESM.zip › MSB-2023-12002R_SourceData_Figure7/MSB-2023-12002R_Source data Figure 7A.tif]

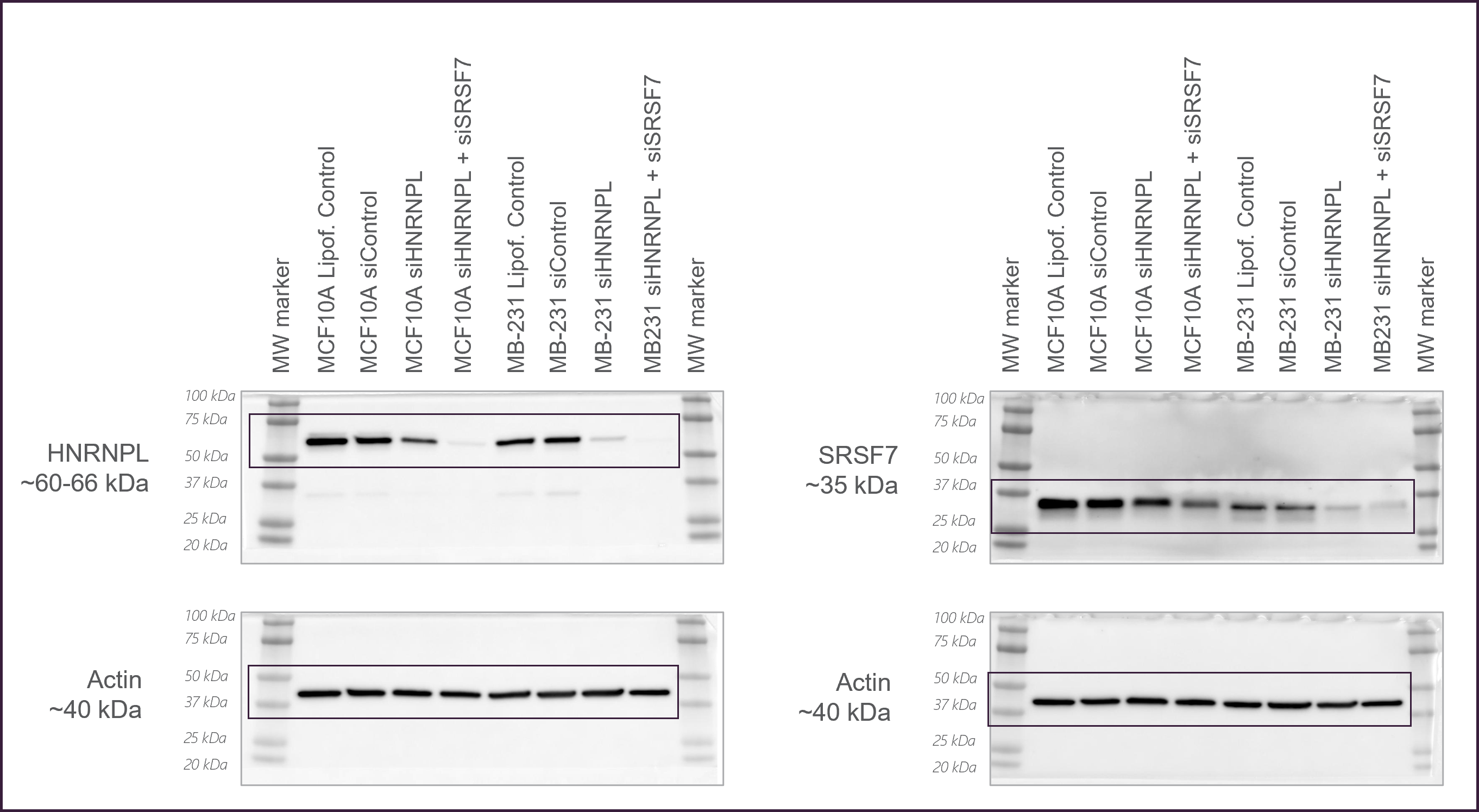

Supplement: Supplementary file 10 — EV Figure Source Data [file 44320_2024_34_MOESM10_ESM.zip › MSB-2023-12002R_SourceData ExpandedView/SourceDataExpandedView3A-Western.tif]

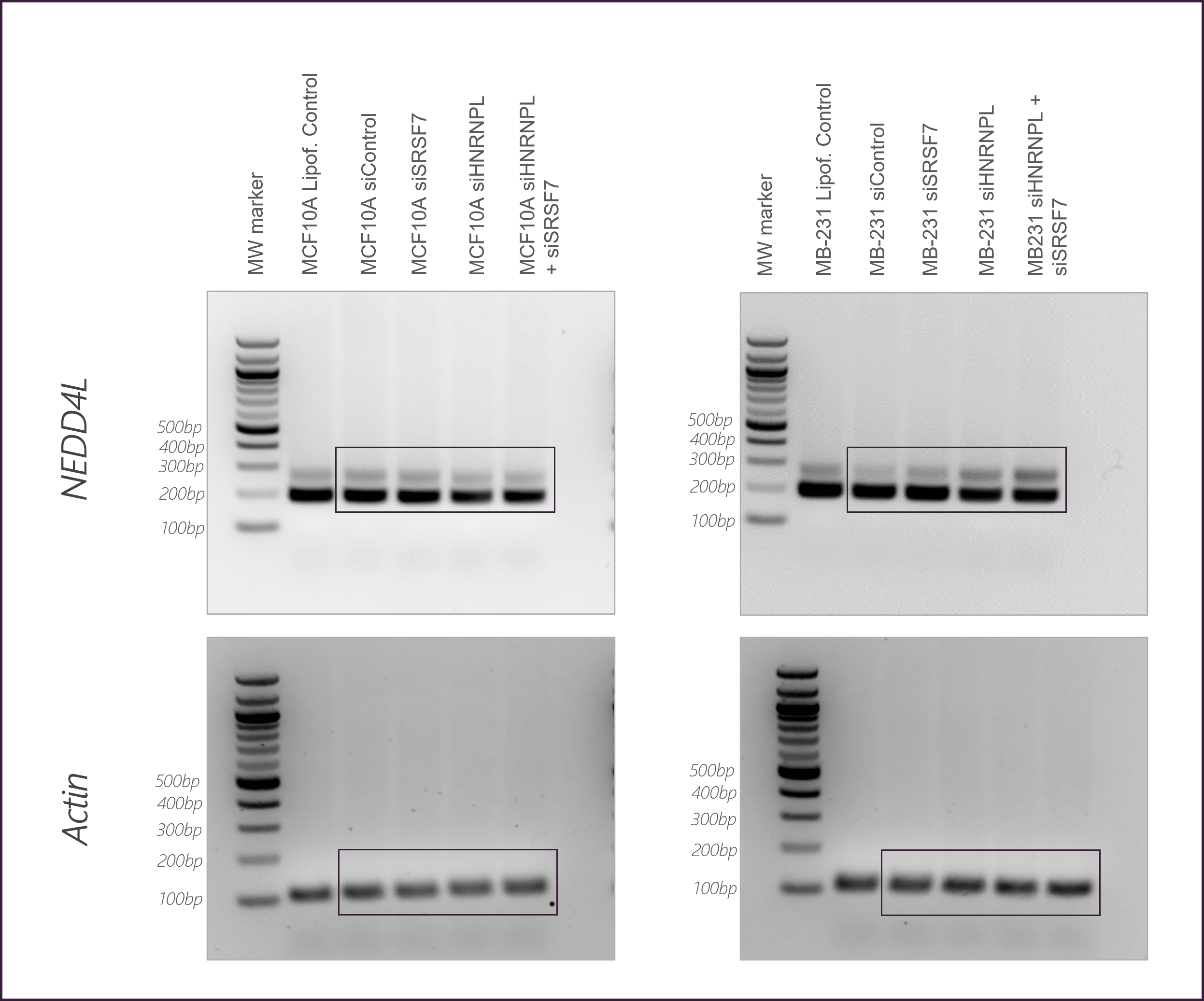

Supplement: Supplementary file 10 — EV Figure Source Data [file 44320_2024_34_MOESM10_ESM.zip › MSB-2023-12002R_SourceData ExpandedView/SourceDataExpandedView3B-PCR.tif]

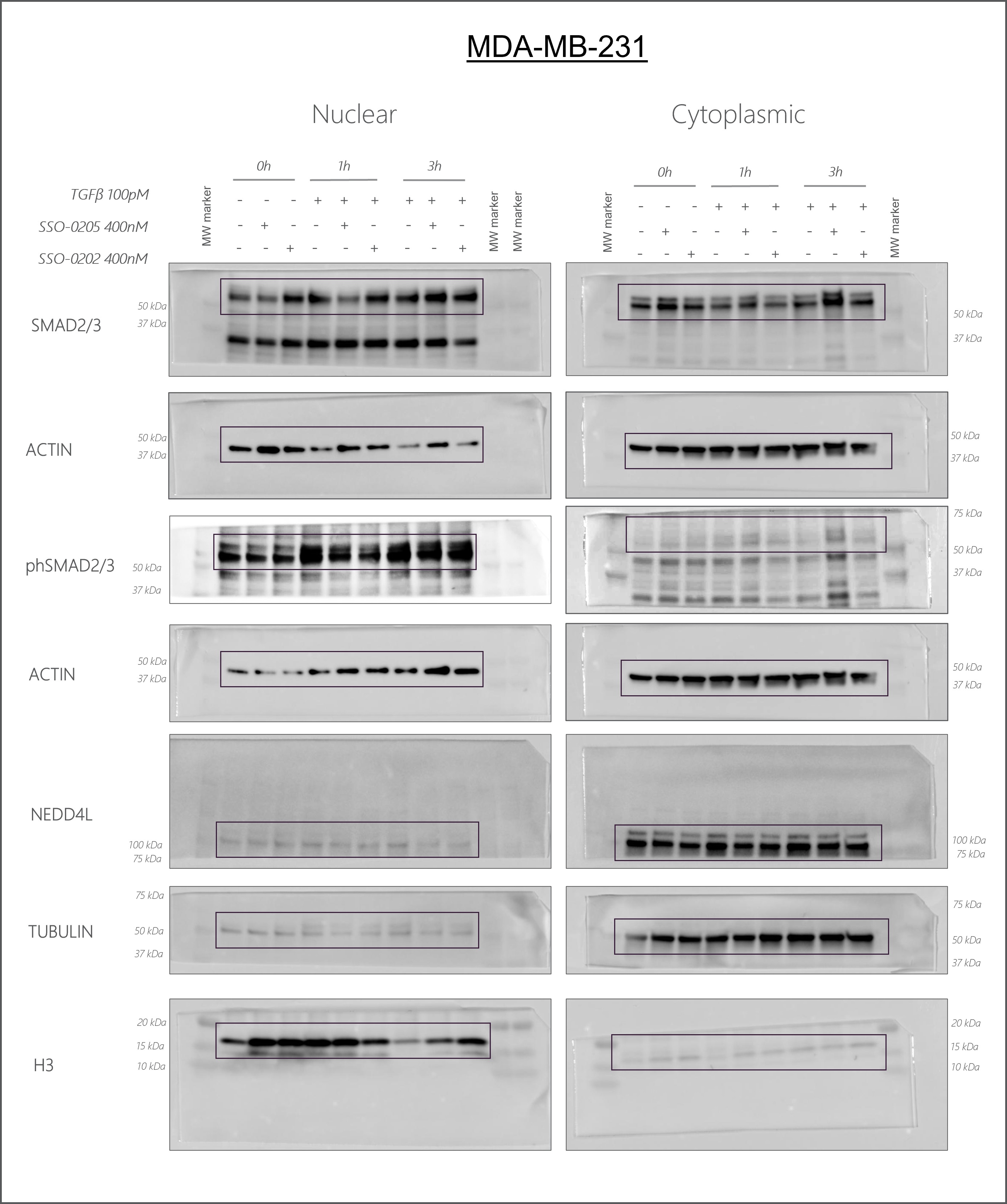

Supplement: Supplementary file 10 — EV Figure Source Data [file 44320_2024_34_MOESM10_ESM.zip › MSB-2023-12002R_SourceData ExpandedView/SourceDataExpandedView4A-Western.tif]

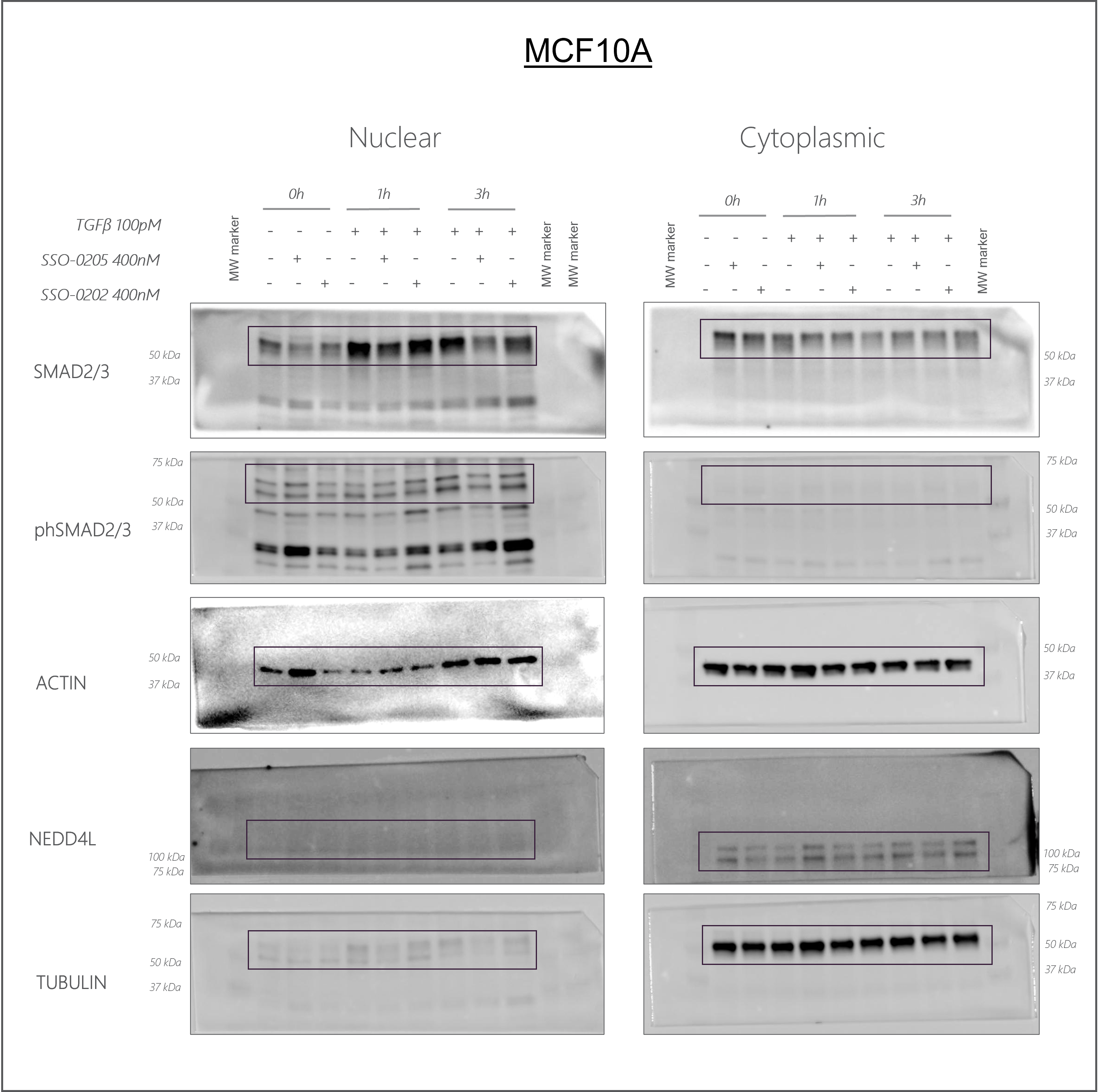

Supplement: Supplementary file 10 — EV Figure Source Data [file 44320_2024_34_MOESM10_ESM.zip › MSB-2023-12002R_SourceData ExpandedView/SourceDataExpandedView4B-Western.tif]

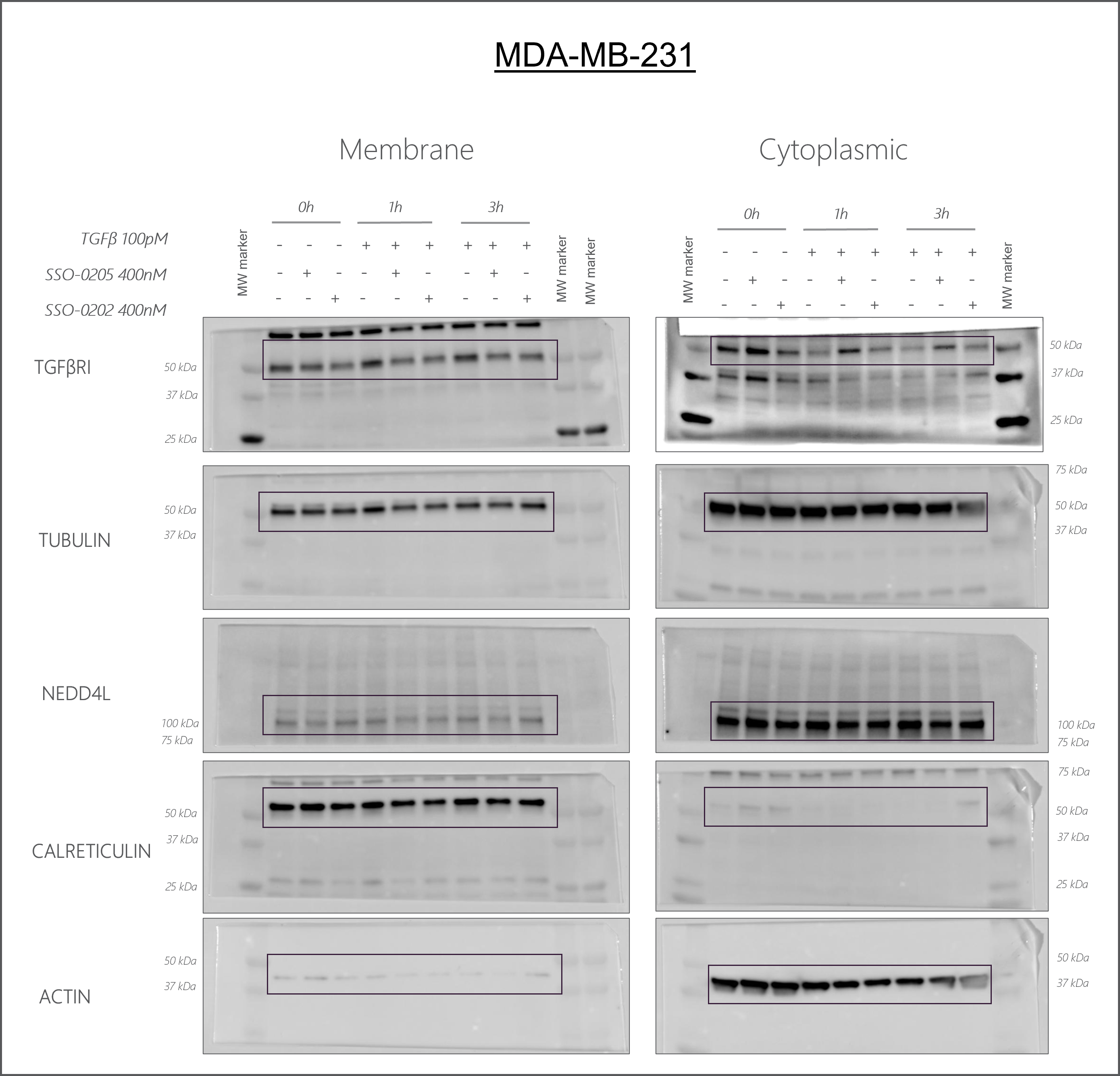

Supplement: Supplementary file 10 — EV Figure Source Data [file 44320_2024_34_MOESM10_ESM.zip › MSB-2023-12002R_SourceData ExpandedView/SourceDataExpandedView4C-Western.tif]

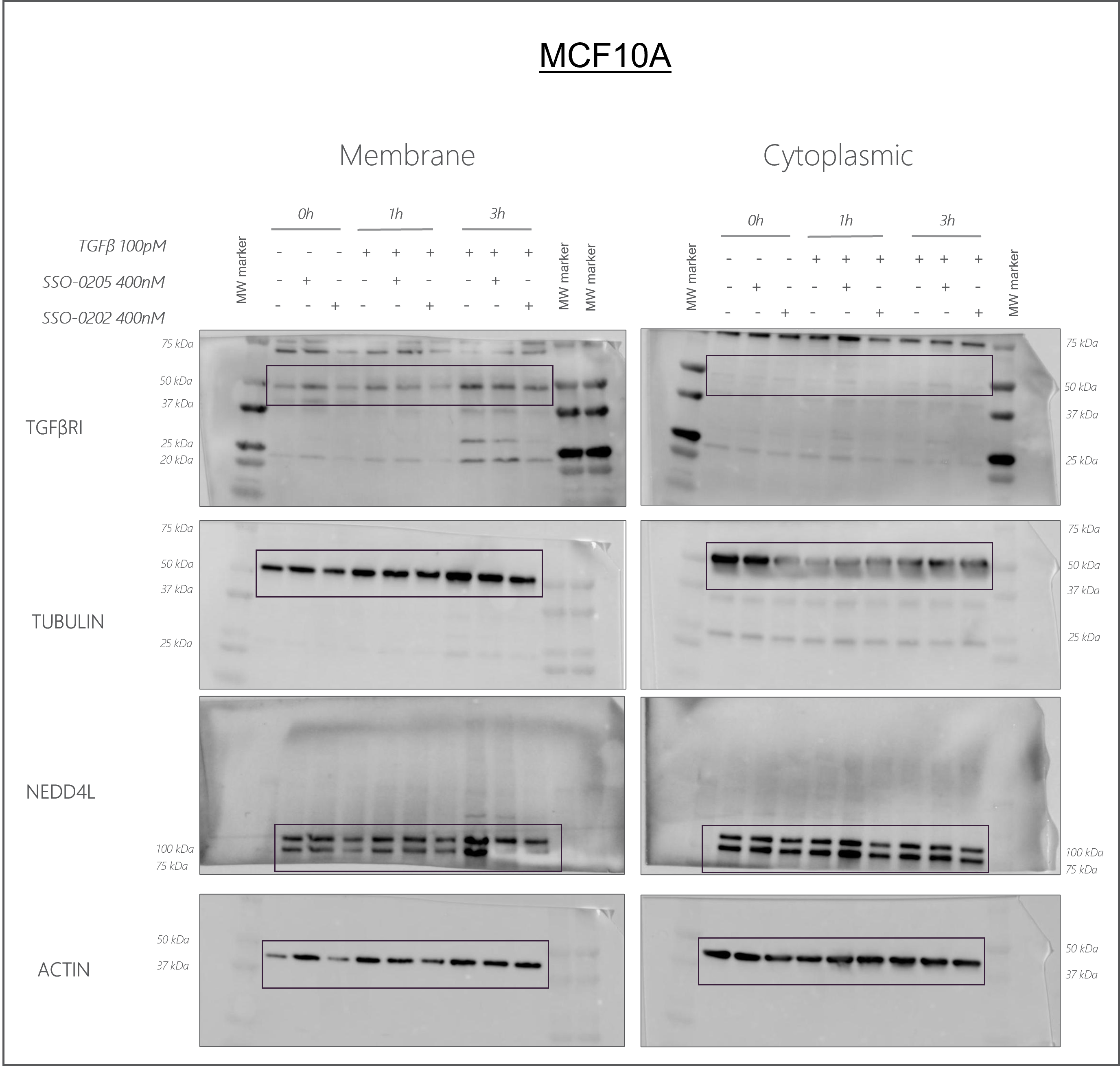

Supplement: Supplementary file 10 — EV Figure Source Data [file 44320_2024_34_MOESM10_ESM.zip › MSB-2023-12002R_SourceData ExpandedView/SourceDataExpandedView4D.tif]
