# Supplementary material for: Development and validation of AI/ML derived splice-switching oligonucleotides
Source: Mol Syst Biol. 2024 Apr 25;20(6):676–701. doi: 10.1038/s44320-024-00034-9 (PMC11148135; doi:10.1038/s44320-024-00034-9)
Supplement: Supplementary file 12 — Expanded View Figures [file 44320_2024_34_MOESM12_ESM.pdf]

Expanded View Figures

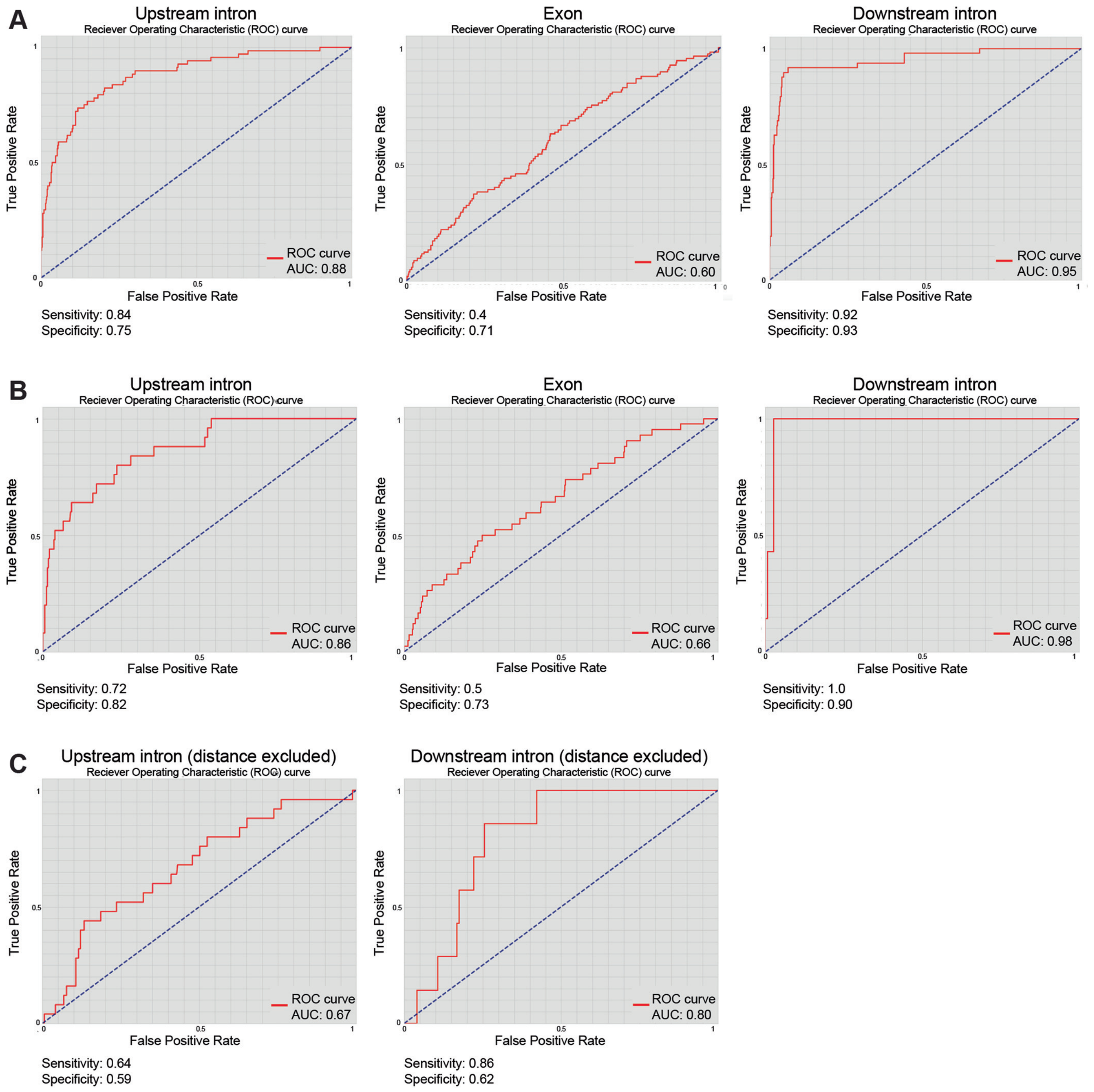

Figure EV1. XGboost training and testing with independent data.

(**A**) ROC curves and sensitivity/specificity calculations for the three different models using the MFASS dataset. (**B**) ROC curves and sensitivity/specificity calculations for the three different models using the Vex-seq dataset. (**C**) ROC curves and sensitivity/specificity calculations for the XGboost-upstream and -downstream models when "distance to splice site" is not included as a feature.

 © The Author(s)

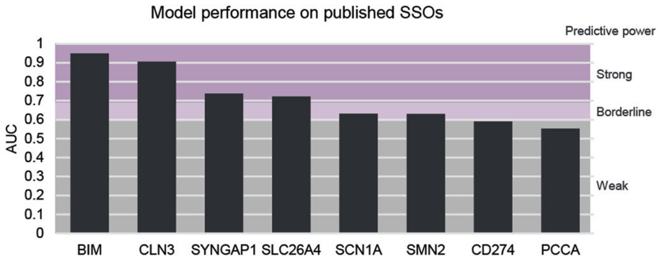

Figure EV2. XGboost model performance on known SSO modulated AS events.

AUC values for each alternative splicing event where both positive and negative SSOs were evaluated in previous publications. Enrichment scores were used as a metric of functional SSO prediction, and positive and negative labels were derived from the publications indicated.

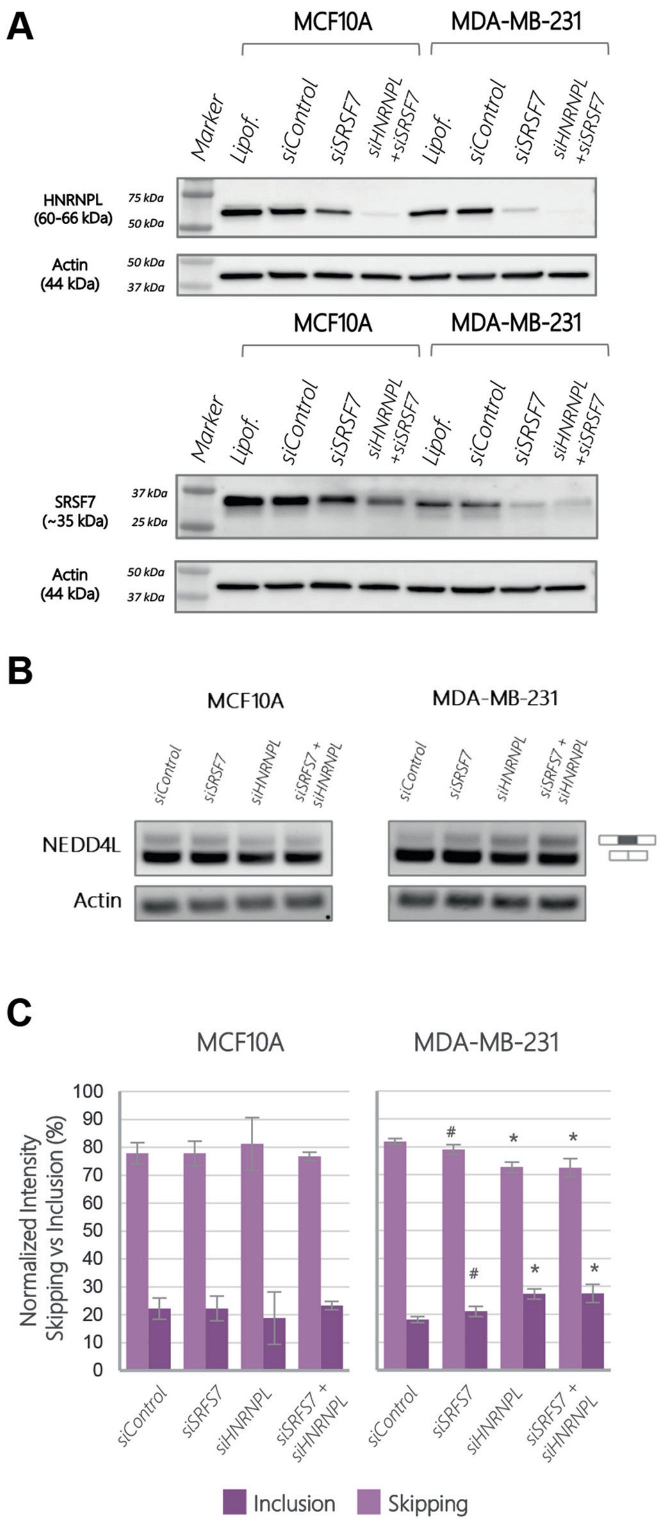

Figure EV3. Kock-down of the HNRNPL and SRSF7 promote NEDD4Le13 inclusion in the TNBC cancer cell line.

(**A**) Western blots measuring siHNRNPL (left panel) and siSRSF7 (right panel) transfection efficiency by protein decreased in MCF10A and MB231 cells treated with corresponding siRNA alone or in combination for 70 h at 20 nM. (**B**) Agarose gel for PCR product measuring *NEDD4L* isoforms in MDA-MB-231 and MCF10A cells in response to siRNA treatments. (**C**) Quantification of agarose gels ($n = 3$ biological replicates). Mean and Standard deviation are represented. Statistical differences between each siRNA treatment group (inclusion or skipping) vs corresponding siControl were calculated by Student's *t*-test; *≤0.05; #≤0.1. Source data are available online for this figure.

 © The Author(s)

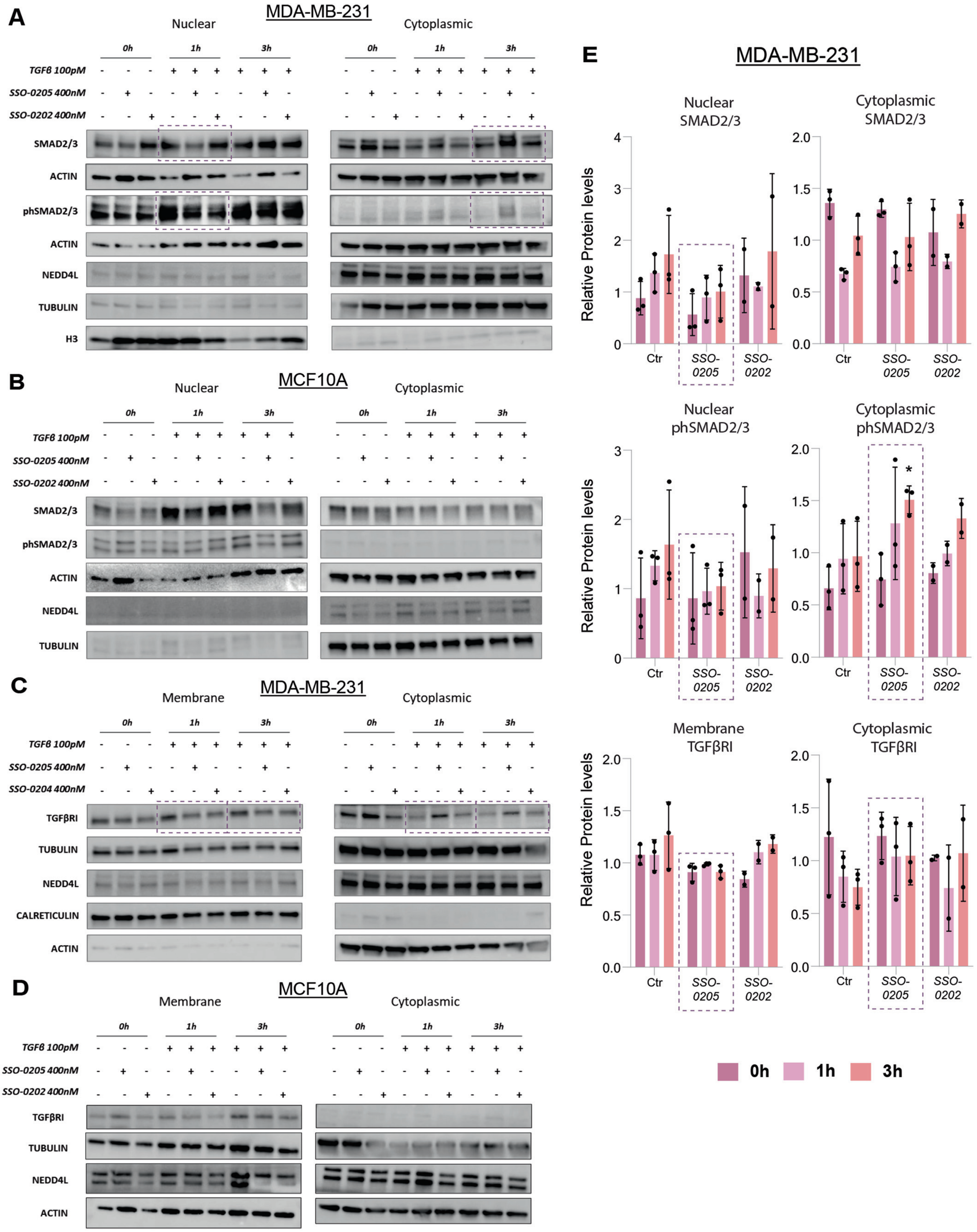
A
MDA-MB-231
Nuclear
Cytoplasmic
0h
1h
3h
TGFβ 100pM
SSO-0205 400nM
SSO-0202 400nM
SMAD2/3
ACTIN
phSMAD2/3
ACTIN
NEDD4L
TUBULIN
H3
B
MCF10A
Nuclear
Cytoplasmic
TGFβ 100pM
SSO-0205 400nM
SSO-0202 400nM
SMAD2/3
phSMAD2/3
ACTIN
NEDD4L
TUBULIN
C
MDA-MB-231
Membrane
Cytoplasmic
TGFβ 100pM
SSO-0205 400nM
SSO-0204 400nM
TGFβRI
TUBULIN
NEDD4L
CALRETICULIN
ACTIN
D
MCF10A
Membrane
Cytoplasmic
TGFβ 100pM
SSO-0205 400nM
SSO-0202 400nM
TGFβRI
TUBULIN
NEDD4L
ACTIN
E
MDA-MB-231
Nuclear SMAD2/3
Cytoplasmic SMAD2/3
Nuclear phSMAD2/3
Cytoplasmic phSMAD2/3
Membrane TGFβRI
Cytoplasmic TGFβRI
Relative Protein levels
Ctr
SSO-0205
SSO-0202
*
0h
1h
3h

Figure EV4. SSO-0205 modulates the TGFβ pathway response in MDA-MB-231 cells.

(**A**,**B**) Western blots measuring TGFβ-pathway related proteins in respective nuclear/cytoplasmic subcellular fractions in response to TGFβ stimulation (0, 3, or 6 h) after SSOs treatment (24 h) in MDA-MB231 (**A**) and MCF10A (**B**) Note that cytoplasmic ACTIN is shared for Cytoplasmic *SMAD2/3 and phSMAD2/3,* since the membrane was stripped and re-probed. (**C**,**D**) Western blots measuring TGFβ-pathway related proteins in respective membrane/cytoplasmic subcellular fractions in response to TGFβ stimulation (0, 3, or 6 h) after SSOs treatment (400 nM) (24 h) in MDA-MB-231 (**C**) and MCF10A (**D**). (**E**) Quantification of MDA-MB-231's Western blots in the respective subcellular locations for SMAD2/3 levels (top panel), phosphorylated SMAD2/3 (phSMAD2/3) levels (middle panel), and TGFβRI (lower panel). ($n = 2$–3 biological replicates). Mean and Standard deviation are represented. Statistical differences calculated by Student's *t*-test vs the corresponding time point at the TGFβ alone group; *≤0.05. Source data are available online for this figure.

© The Author(s)
